# Type 2 diabetes-associated carotid plaque burden is increased in patients with retinopathy compared to those without retinopathy

**DOI:** 10.1186/s12933-015-0196-1

**Published:** 2015-03-18

**Authors:** Núria Alonso, Alicia Traveset, Esther Rubinat, Emilio Ortega, Nuria Alcubierre, Jordi Sanahuja, Marta Hernández, Angels Betriu, Carmen Jurjo, Elvira Fernández, Didac Mauricio

**Affiliations:** Department of Endocrinology and Nutrition, Health Sciences Research Institute and University Hospital Germans Trias i Pujol, Badalona, Spain; Department of Ophthalmology, Hospital Universitari Arnau de Vilanova, Lleida, Spain; Department of Endocrinology and Nutrition, Hospital Universitari Arnau de Vilanova, Lleida, Spain; Institut de Recerca Biomedica de Lleida, University of Lleida, Lleida, Spain; Department of Endocrinology and Nutrition, Institut d’Investigacions Biomediques August Pi Suñer, CIBER de Diabetes y Enfermedades Metabólicas asociadas, Hospital Clinic, 08036 Barcelona, Spain; Unitat de Detecció i Tractament de Malalties Aterotrombòtiques, Hospital Universitari Arnau de Vilanova, Lleida, Spain; Department of Neurology, Hospital Universitari Arnau de Vilanova, Lleida, Spain; Department of Nephrology, Hospital Universitari Arnau de Vilanova, Lleida, Spain

**Keywords:** Type 2 diabetes, Cardiovascular disease, Retinopathy, Carotid plaque

## Abstract

**Background:**

Cardiovascular disease (CVD) is the leading cause of mortality among subjects with type 2 diabetes (T2D), and diabetic retinopathy (DR) has been associated with an increased risk for CVD. The present study was designed to test the concept that T2D patients with DR, but without previous cardiovascular (CV) events and with normal renal function, have an increased atherosclerotic burden compared with patients without DR.

**Methods:**

A cross-sectional study was performed using patients with normal renal function (estimated glomerular filtration rate (eGFR) >60 ml/min) and without previous CV events. A total of 312 patients (men, 51%; mean age, 57 yrs; age range 40–75 yrs) were included in the study; 153 (49%) of the patients had DR. B-mode carotid ultrasound imaging was performed for all of the study subjects to measure the carotid intima-media thickness (cIMT) and carotid plaques in the common carotid artery (CCA), bifurcation and internal carotid artery (ICA).

**Results:**

The percentage of carotid plaques in T2D patients with DR was higher than in T2D patients without DR (68% vs. 52.2%, p = 0.0045), and patients with DR had a higher prevalence of ≥2 carotid plaques (44.4% vs. 21.4%; *p* < 0.0001). No differences were observed in the cIMT measured at different carotid regions between the patients with or without DR. Using multivariate logistic regression (adjustment for major risk factors for atherosclerosis), DR was independently associated with mean-internal cIMT (*p* = 0.0176), with the presence of carotid plaques (*p* = 0.0366) and with carotid plaque burden (≥2 plaques; *p* < 0.0001).

**Conclusions:**

The present study shows that DR in T2D patients without CVD and with normal renal function is associated with a higher atherosclerotic burden (presence and number of plaques) in the carotid arteries. These patients may be at a higher risk for future CV events; therefore, an ultrasound examination of the carotid arteries should be considered in patients with DR for more careful and individualised CV assessment and follow-up.

## Background

The deleterious effects of hyperglycaemia are classically separated into microvascular (retinopathy, diabetic nephropathy and neuropathy) and macrovascular complications (coronary artery disease, peripheral arterial disease and cerebrovascular disease).

Traditionally, the micro- and macrovascular complications of diabetes have been viewed, studied, and treated as distinct and independent disorders. However, accumulating data from epidemiological and pathophysiological studies suggest that these vascular complications may share common pathophysiological mechanisms beyond those related to traditional cardiovascular (CV) risk factors. Data obtained from epidemiological studies have clearly demonstrated that diabetic retinopathy (DR), a common chronic microvascular complication of diabetes, is associated with macrovascular disease [1] as well as with increased CV morbidity and mortality in patients with type 2 diabetes mellitus (T2D) [2]. In these patients, the presence of DR has been described as an independent risk factor for incident coronary heart disease [3,4] and ischaemic stroke [5]. Among the possible mechanisms described to explain the interconnection between diabetic micro- and macroangiopathy are metabolic disturbances [6]. Other authors have suggested that microangiopathy of the vasa vasorum (VV), the plexus of microvessels that partially provides oxygen and nutrients to the walls of large arteries, may be involved in diabetic atherosclerosis [7,8]. Our group recently reported that T2D patients with DR had increased angiogenesis of the VV of the common carotid artery (CCA) [9]. These findings were recently confirmed by Sampson *et al.* in a larger study of T2D patients [10].

When a clinical CV event occurs, atherosclerotic disease is difficult to reverse. In these cases, ultrasonography of the carotid arteries is frequently used to detect early signs of atherosclerosis, i.e., increased thickness of the arterial wall or the occurrence of plaques. Ample data suggest that plaque and carotid intima-media thickness (cIMT) are associated with prevalent and incident coronary heart disease (CHD) and stroke, with the presence of plaque generally having a stronger association with CVD compared to cIMT alone [11]. Thus, it has recently been demonstrated that ultrasound assessment of a carotid plaque and its total volume or total area progression is a stronger predictor of future CV events than cIMT measurement [12,13]. A high prevalence (between 43% and 64%) of carotid plaques has been described in T2D patients without evidence of CV disease (CVD) [14-16]. Prospective studies conducted in T2D patients free of any CV event have shown that the percentage of patients with carotid artery plaques is higher in those who develop a CV event compared with those who are free from CV events [15,16]. Recent epidemiological studies have demonstrated that chronic kidney disease (CKD), defined as an estimated glomerular filtration rate (eGFR) <60 ml/min, is an independent risk factor for atherosclerotic disease [17] and for the presence of carotid plaques [18]. In T2D patients, a high prevalence of low eGFR without associated albuminuria has been reported and described to be associated with atherosclerosis of peripheral arteries, independently of albuminuria, [19] and with cIMT in several [20], but not all, studies [21,22].

To test the concept that patients with T2D and DR have an increased prevalence of subclinical atherosclerosis, as shown using carotid plaque burden and cIMT measurements, we investigated the association between these measures and retinopathy in a group of patients with T2D but without previous CVD and with normal renal function.

## Methods

### Subjects

T2D patients were selected to participate in this single-centre study, which aimed to investigate the association between carotid atherosclerosis and DR in T2D. All study participants were recruited from the outpatient clinic at our centre, and potential candidates were identified by screening patients enrolled in the diabetic eye disease programme of our centre as well as those visiting the diabetic retinopathy unit. The inclusion criteria for both groups were as follows: age range, 40–75 yrs; absence of established impaired renal function (calculated glomerular filtration rate (eGFR) <60 ml/min); and known CVD. From the total number of subjects who were initially recruited, 60 subjects were excluded after the screening visit because of the presence of an exclusion criterion (previous CVD, impaired renal function, increased urine albumin excretion, or not having T2D), and 15 additional patients withdrew from the study after the initial screening visit (consented withdrawal before completing the study assessment). A total of 312 participants with (n = 153) or without (n = 159) DR were included in the study. To achieve a uniform age distribution, patients with and without DR were selected to have representative and similar numbers of patients by gender and age (stratified according to 5-yr age intervals). However, we could not recruit enough patients with retinopathy in the age range between 40 and 50 yrs. For the purpose of this study, a patient was arbitrarily considered to have previous hypertension or dyslipidaemia if the patient was taking medication for the given condition. The weight, height and waist circumference of the subjects were measured using standardised methods, and the blood pressure (mean of 2 measurements, 5 min apart) of the subjects was measured after 10 min in the seated position using a blood pressure monitor (HEM-7001E, Omron, Barcelona, Spain). The patients underwent a complete eye evaluation by experienced ophthalmologists (AT and CJ) to assess the presence or absence of diabetic retinopathy according to an international clinical DR consensus [23]. Retinopathy was evaluated using multifield stereoscopic retinal photography with the following definitions: a) mild nonproliferative DR - microaneurysms only; b) moderate nonproliferative DR - more than just microaneurysms but less than severe nonproliferative DR; c) severe nonproliferative DR by any of the following - more than 20 intraretinal haemorrhages in each of 4 quadrants, definite venous beading in 2+ quadrants, prominent intraretinal microvascular abnormalities in 1+ quadrant, and no signs of proliferative retinopathy; and d) proliferative DR, by neovascularisation and/or vitreous/preretinal haemorrhage. In addition to the anamnestic evaluation and physical examination, the patients’ clinical records were reviewed to rule out any previously known CV events or associated revascularisation procedures, including coronary heart disease, cerebrovascular disease, or peripheral vascular disease (including the diagnosis of diabetic foot disease). Ours is the only reference hospital for CVDs and procedures in the region, but we also had access to reports from three small hospitals in the region. Therefore, along with the anamnestic evaluation, we could ascertain any CV events that occurred in the health-care area. Heart failure and macroalbuminuria (urine albumin/creatinine ratio >299 mg/g) were also considered exclusion criteria. Serum and spot urine samples were collected in the fasting state, and all serum and urine tests were performed using standard laboratory methods. Low-density lipoprotein cholesterol was estimated using the Friedewald formula, and eGFR was estimated using the diet modification in renal disease (MDRD-4) formula [24]. Haemoglobin A_1c_ (HbA_1c_) levels were determined using HPLC (Variant^TM^, Bio-Rad Laboratories SA, Spain), and its concentrations are expressed in National Glycohemoglobin Standardization Program/Diabetes Control and Complications Trial units. Urine albumin was measured using an immunoturbidimetric method and a Roche/Hitachi Modular P analyser (Roche Diagnostics, Spain).

### Carotid ultrasound imaging study

All of the study participants underwent the same carotid ultrasound protocol. All measures and ultrasound studies were assessed by the same researcher (ER), who was blinded to the conditions of the participants and did not have access to the study data. B-mode ultrasound imaging was performed using a Siemens Sequoia 512 and a 15-Mhz linear array probe. Moreover, a standardised imaging protocol was performed to evaluate intima-media-adventitia thickness (IMT), defined as the distance between the lumen-intima and the media-adventitia ultrasound interfaces (intima-media complex), and plaque presence in the carotid arteries. The patients were examined from their back; they were placed in the supine position with the head turned 45° contralateral to the side of scanning. Images were obtained in longitudinal sections, with a single lateral angle of insonation and optimisation of the image to the far wall. The B-mode images of the left and right segments were recorded and electronically stored. The last (previous to the bulb) and first (starting at the flow divider) centimetres of the common and internal carotid arteries, respectively, and the total length of the bulb were used for IMT measurements, which were performed off-line using semiautomatic software. The data (mean IMT and mean-maximum IMT) from each segment were provided, and the right- and left-side values were averaged to obtain the mean and mean-maximum common carotid (CC), carotid bulb, and internal carotid (ICA) measurements. Plaques were identified using B-mode and colour Doppler examinations in both the longitudinal and transverse planes to consider circumferential asymmetry and were defined as a “focal structure that encroaches into the arterial lumen of at least 0.5 mm or 50% of the surrounding cIMT value or demonstrates a thickness of 1.5 mm, as measured from the media adventitia interference to the intima-lumen surface” according to the Mannheim consensus [25].

The local Ethics Committee of Hospital Arnau de Vilanova (Lleida, Spain) approved the protocol, and all of the patients signed written informed consent forms.

### Sample size

To determine the sample size, we used preliminary data from a previous study of our group in which the plaque frequency was 69% in patients with retinopathy or 52% in patients without retinopathy [10]. We calculated a sample size of 126 subjects for each study group, which would allow an 80% power to detect differences between groups with a significance level of <0.05. Thus, the number of patients included in each group was sufficient to test the initial hypothesis.

### Statistical analysis

The data are presented as the median values and 25th and 75th percentiles, means ± standard deviations (SDs), and n (%), as appropriate. Non-normally distributed variables were log transformed to reduce skewness, and normality was re-evaluated. Between-group (DR vs. non-DR, male vs. female, and with hypertension vs. without hypertension, or dyslipidaemia, or smoking habit) differences in anthropometric, clinical, cIMT, and laboratory variables were evaluated using the chi-squared test (for categorical variables), Wilcoxon test (for continuous, non-normally distributed variables), or Student’s *t*-test (for continuous, normally distributed variables). Unadjusted and adjusted general linear models (PROC GLM in SAS) were used to test whether cIMT values differed between diabetic patients with retinopathy vs. those without retinopathy. Univariate or multiple logistic regression models were used to evaluate the differences in plaque presence between the groups. The following factors were entered into the adjusted general linear and multiple logistic regression models: age, sex, body mass index, presence of hypertension, presence of dyslipidaemia, smoking habit, urinary albumin excretion rate and eGFR. The significance level was defined as *p* ≤ 0.05, and all analyses were performed using SAS software, v. 9.2 (SAS Institute Inc., USA).

## Results

### Clinical variables

Among the 312 study patients with T2D, 153 (49.03%) had DR. The patients with retinopathy were older due to difficulties recruiting patients with DR who were aged <50 yrs. As typically observed in previous studies comparing patients with and without DR, the patients with this condition had longer diabetes duration and were more likely to receive insulin treatment and antiplatelet agents. These patients also exhibited a higher prevalence of hypertension, higher systolic blood pressure, and increased waist circumference, HbA1c level, HDL-c level, and urinary albumin/creatinine ratio compared with those without DR (Table 1). The distribution of retinopathy grades was as follows: 60 patients had mild retinopathy (39.2%), 57 patients had moderate retinopathy (37.3%), 29 patients had severe nonproliferative retinopathy (18.9%), and 7 patients had severe proliferative retinopathy (4.6%).

**Table 1:** **Characteristics of the study groups**

	**Diabetes without retinopathy (n = 159)**	**Diabetes with retinopathy (n = 153)**	***p***
Sex (male/female)	82/77	76/77	0.7373
Non-Caucasian	7 (4.4%)	6 (4.6%)	0.9413
Age (yr)	59 (48–66)	61 (54–68)	0.0127
Disease duration (yr)	6 (2–10)	11 (6–20)	<0.0001
Insulin treatment (n)	20 (12.6%)	84 (54.9%)	<0.0001
Smoking (current/past/never)	71/54/32	78/44/30	0.5134
Antiplatelet agents	112/47 (29.6%)	82/71 (46.4%)	0.0022
Dyslipidaemia (yes/no)	88/71 (44.7%)	79/74 (48.4%)	0.5111
Hypertension (yes/no)	83/76 (47.8%)	48/105 (68.3%)	0.0002
Systolic BP (mm Hg)	134 (123–145)	143 (133–159)	<0.0001
Diastolic BP (mm Hg)	76 (70–83)	77.5 (68.5-85.5)	0.7526
HR (b/min)	75 (68–82)	77 (70–86)	0.0767
Body mass index (kg/m^2^)	30.3 (27.4-34.0)	31.1 (28.3-35)	0.2477
Waist circumference (cm)	103 (96–111)	107.5 (163–114)	0.0039
Haemoglobin A_1c_ (%)	7.1 (6.5-7.9)	8.1 (7.2-9.1)	<0.0001
Total cholesterol (mmol/l)	184 (163–207)	181 (162–204.5)	0.9889
HDL-cholesterol (mmol/l)	47 (40–57)	50.5 (42–60.5)	0.0308
LDL-cholesterol (mmol/l)	108 (90.2-130.2)	105.4 (86.5-127.8)	0.2694
Triglycerides (mmol/l)	118 (89–171)	116 (83–168)	0.6181
Serum creatinine (μmol/l)	0.79 (0.69-0.92)	0.79 (0.68-0.93)	0.7975
Urinary albumin/creatinine ratio, mg/g	5.8 (3.2-11)	12.4 (6–32.7)	<0.0001

Data are presented as the median values and interquartile ranges. BP, blood pressure.

### Ultrasound examination

The mean and mean-maximum cIMT at different territories (common, bulb, and internal carotid) did not differ between the patients with DR and those without DR, except that a higher mean-maximum common cIMT was observed in the patients with DR (*p* = 0.0187); these differences disappeared after adjustment (Table 2). The general linear models were used to evaluate the cIMT determinants. Age (*p* < 0.0001) and active smoking (*p* = 0.0641); age (*p* = 0.0154) and hypertension (*p* = 0.0140); and age (*p* = 0.0131), sex (*p* = 0.0327), and the presence of retinopathy (*p* = 0.0176) were independently associated with mean CCA-IMT, bulb-IMT, and ICA-IMT, respectively. No association was found between any of the cIMT measurements at different territories or between the UAE and eGFR values or diabetes duration.

**Table 2:** **Results of the ultrasonographic carotid study: carotid intima-media thickness and presence of carotid plaques**

	**No retinopathy (n = 159)**	**Retinopathy (n = 153)**	**P value**
Mean common cIMT	0.789 (0.686-0.889)	0.802 (0.731-0.907)	0.1474
Mean-max common cIMT	0.941 (0.820-1.049)	0.986 (0.889-1.073)	0.0187
Mean bulb cIMT	0.835 (0759–0.955)	0.861 (0.723-0.928)	0.6674
Mean-max bulb	1.047 (0.922-1.199)	1.007 (0.919-1.227)	0.7470
Mean-internal cIMT	0.677 (0-566-0.782)	0.623 (0.534-0.713)	0.1151
Mean-max internal cIMT	0.845 (0.704-1.009)	0.810 (0.685-0.909)	0.2070
Carotid plaques, n (%)	83 (52.2%)	104 (68%)	0.0045
Carotid plaques (none/1/>1) (n)	76/49/34	49/36/68	<0.0001
Common carotid plaque, n (%)	9 (10.8%)	19 (18.3%)	0.1574
Bulb plaque, n (%)	67 (80.7%)	89 (85.6%)	0.3752
Internal carotid plaque, n (%)	30 (36.1%)	51 (49%)	0.0771

The percentage of patients with carotid plaques was higher in those with DR compared to those without DR (68% vs. 52.2%, p = 0.0045), and of these patients, the DR group contained more patients with ≥2 carotid plaques (44.4% vs. 21.4%, *p* < 0.0001). No association was found between carotid plaque burden (≥2 carotid plaques) and the duration of diabetes. In relation to the territory, there was a tendency towards a higher frequency of plaques at the internal carotid artery (ICA) in the group of patients with DR (36% vs. 49%, p = 0.07). The variables that were independently associated with the presence of carotid plaques were age (p = 0.0022), hypertension (p = 0.0300), the presence of retinopathy (p = 0.0366), and active smoking (p = 0.0109) (Table 3). The variables that were associated with the presence of ≥2 carotid plaques were age (p < 0.0001), sex (p = 0.0015), dyslipidaemia (p = 0.0031), and the presence of retinopathy (p < 0.0001) (Table 4).

**Table 3:** **Multivariate logistic regression for the presence of carotid plaques**

	**Odds ratio (95% confidence interval)**	**P value**
Age	1.04 (1.01-1.07)	0.0022
BMI	0.98 (0.93-1.03)	0.4578
Female sex	0.79 (0.47-1.34)	0.3952
DR	1.71 (1.03-2.85)	0.0366
Hypertension	1.78 (1.05-3.00)	0.0300
Dyslipidaemia	1.16 (0.71-1.89)	0.5505
Smoking	2.17 (1.13-4.14)	0.0109
eGFR	1.00 (0.99-1.01)	0.3345
UAE	0.99 (0.99-1.00)	0.6397

DR: diabetic retinopathy; UAE: urinary albumin excretion rate; eGFR: estimated glomerular filtration rate.

**Table 4:** **Multivariate logistic regression for carotid plaque burden (≥2 plaques)**

	**Odds ratio (95% confidence interval)**	**P value**
Age	1.09 (1.05-1.13)	<0.0001
BMI	0.98 (0.92-1.04)	0.6012
Female sex	0.36 (0.19-0.67)	0.0015
DR	3.17 (1.75-5.75)	<0.0001
Hypertension	2.00 (1.08-3.70)	0.0255
Dyslipidaemia	2.32 (1.33-4.05)	0.0031
Smoking	1.62 (0.77-3.39)	0.1980
eGFR	1.00 (0.99-1.02)	0.3177
UAE	0.99 (0.98-1.00)	0.3107

DR: diabetic retinopathy; UAE: urinary albumin excretion rate; eGFR: estimated glomerular filtration rate.

No differences in cIMT measurements or in plaque presence were observed among the groups with different grades of retinopathy (mild vs. moderate/severe/proliferative).

## Discussion

The present study shows that T2D patients with DR who are free of clinical CVD and with normal renal function have increased atherosclerotic burden in their carotid arteries compared with patients without DR.

Epidemiological studies have demonstrated that DR in T2D patients is associated with a 1.7-fold increased risk of CV events, such as stroke, coronary artery disease and heart failure [26]. Notably, this risk persists after adjusting for traditional CV risk factors, diabetes duration and diabetes control, suggesting that microvascular disease may contribute to the development of CVD in diabetes. A major finding of the present study is that DR is independently associated with the presence of carotid plaques. This result is similar to that observed in the study by Kreutzenberg *et al.*, in which DR was independently associated with carotid plaques, although the number of patients with retinopathy was lower in that study than in our study, and patients with previous CV events were not excluded [27]. On the other hand, several studies have described an association between cIMT thickness, as a surrogate marker of atherosclerosis, and DR that is independent of traditional risk factors and glucose control [28,29]. Miyamoto *et al.* also reported an association between common carotid arterial diameter, as a marker of atherosclerosis, and the presence of DR [30]. It should be noted that in the present study, the percentage of patients with carotid plaques was not only higher in those with DR than in those without DR but that the number of carotid plaques was also elevated. To the best of our knowledge, there is no report in the literature regarding atherosclerotic plaque burden in T2D patients with or without DR. A direct association has been described between a plaque score, consisting of the sum of the maximum plaque thicknesses of different carotid territories, and the presence and extent of coronary artery disease in T2D patients [31]. As reported in general-population studies, the risk of CV events, such as stroke and cerebral infarction, gradually increases with incremental total plaque number, irrespective of the location of the plaques [32]. Moreover, carotid plaque measurements have been shown to be more strongly predictive of CV events than cIMT measurements. The extent of atherosclerosis in the carotid artery, measured either as total plaque area [30] or as progression of total plaque volume [12], has been shown to be an independent predictor of coronary events in patients with [12] and without previous CV events [33]. Although these studies included a small proportion of subjects with diabetes, no specific data on these subjects were provided. In the Rotterdam study, a direct relationship between the number of carotid territories with a carotid plaque and incident cases of myocardial infarction was described [34].

Carotid plaque predominantly occurs at sites of nonlaminar turbulent flow, such as the carotid bulb and the proximal ICA, but rarely in the CCA, except in advanced atherosclerotic disease [35]. In the present study, the percentage of plaques in the ICA was higher in T2D patients with DR than in those without DR; however, these differences were not significant. The presence of plaques in the ICA territory is now accepted to be associated with an increased risk of stroke (annual risk of 0.1% to 1.9% for an ICA stenosis <75% to 80%, and annual risk of 2.0% to 3% with greater degrees of stenosis) [36]. Thus, considering research results suggesting that CV events increase as the burden of atherosclerosis increases, we hypothesise that T2D patients with DR and >1 carotid plaque will have a higher risk of CV events. This speculation can only be answered by conducting a prospective study assessing the future occurrence of CV events in these patients.

As stated in almost all studies on DR, the condition is closely related to duration of the disease, higher blood pressure and a higher albumin excretion rate, all of which may contribute to the increased atherosclerosis in these patients [37]. In the present study, these factors were also more frequent in patients with DR. However, of these variables, only hypertension proved to be independently associated with carotid plaques.

It is now accepted that CKD, defined as an eGFR of <60 ml/min, is an independent risk factor for atherosclerotic diseases and is associated with the presence of a carotid plaque independent of albuminuria [38]. Recent studies have emphasised that a large number of T2D patients with low eGFR have a normal UAE [39,40]. It should be noted that little information regarding renal function has been reported in those studies that analysed subclinical atherosclerosis in patients with T2D. In this respect, the study by Saif *et al.*, conducted inT2D patients with normal renal function, did describe a correlation between DR and cIMT [29].

Another ultrasound measure that is used as a surrogate marker for atherosclerosis is cIMT. This measure, as described for carotid plaques, has been reported to be associated with traditional and newer risk factors for atherosclerosis. The cIMT baseline measures and its progression during follow-up are described to be strongly associated with CVD events, even after adjustment for traditional risk factors [41]. cIMT has typically been measured in the CCA, rather than in the carotid bulb or ICA, because high-level measurement precision is easily obtained from this artery. However, it should be noted that the accuracy of cIMT as a marker of atherosclerosis has been questioned by the fact that medial hypertrophy or intimal thickening of CCA may be influenced by factors that do not necessarily reflect the atherosclerotic process, such as age and hypertension [42]. In the present study, T2D patients with DR had a higher mean-maximum common cIMT than did those without DR, whereas no differences were observed in the bulb-cIMT or in the internal cIMT measures between the groups. However, DR was independently associated with only the mean-internal cIMT. In relation to cIMT, it is accepted that, clinically, the measurement of CCA-cIMT is easier and more reliable compared to ICA-cIMT. In contrast, ICA-cIMT appears to predict atherosclerotic cardiac events better than common cIMT [43]. Thus, including internal cIMT and traditional CV risk factors, as well as the presence of plaque in the internal territory, improves the risk classifications of stroke and coronary heart disease in general population studies.

The association between DR and carotid ultrasound measurements has been analysed in large-population cohort studies conducted on subjects without a history of clinical CVD. The ARIC study found that the severity of retinopathy was associated with carotid artery intima-media wall thickness [44], while the CHS [45] and MESA [46] studies found no association between DR and cIMT or carotid stenosis. Possible explanations for the different results obtained in these last two studies and ours may be due to the difference in ethnic backgrounds of patient groups, different criteria used for the definition of subclinical atherosclerosis, different population selection or the absence of bulb territory (a site with a high prevalence of plaques) in the ultrasound examination in these studies. In the present study, no differences in cIMT measurements or in plaque presence were observed among the groups with different grades of retinopathy. This finding is in contrast to the findings of Kreutzenberg *et al.* [27], who described that the retinopathy stage was associated with carotid atherosclerosis. Additionally, in the ARIC study, the severity of retinopathy was associated with cIMT. However, in contrast to the present study, patients with previous CV events were not excluded in those studies. Moreover, the absence of differences observed in cIMT measurements or in plaque presence in relation to retinopathy grades in the present study may be due to few patients in the group having more severe retinopathy. Thus, any conclusion drawn from these results must be made with caution.

In relation to retinopathy and the future risk of CV events, retinopathy has been described in population-based samples as a risk factor (independent from diabetes) for stroke [47], heart failure [48] and coronary heart disease [49]. Patients from the ARIC study who had better CV health, according to the American Heart Association criteria, had a lower prevalence of retinopathy [50]. Moreover, the presence of retinopathy and retinal microvascular signs has recently been described to be associated with an increased risk of cerebral microvascular disease [51].

The strength of the present study is its specific design, which aimed to test the hypothesis that T2D patients with retinopathy have a higher atherosclerotic burden compared with T2D patients without retinopathy. Thus, a large number of patients with retinopathy were included. Only those patients with an eGFR >60 ml/min were included to avoid the confounding factor of renal insufficiency, which is associated with an increased risk of atherosclerosis.

The major limitation of the present study is that a direct relationship between retinopathy and macrovascular disease cannot be demonstrated due to the cross-sectional nature of the study. To assess the CV risk associated with a microvascular complication, such as DR in patients with T2D, longitudinal studies are warranted. To properly assess the CV risk, future studies should include T2D patients with DR (with and without carotid plaques) and T2D patients without DR and other microvascular complications.

## Conclusions

The present study, which has been specifically designed to test the hypothesis that T2D patients with DR who are free of previous CVD and have normal renal function have a higher atherosclerotic burden than those without DR, shows an association between DR and subclinical carotid atherosclerosis. More specifically, T2D patients with DR have an increased atherosclerotic burden (presence of plaques and number of plaques) in the carotid territory. Previous studies performed on the general population have shown that individuals with an increased atherosclerotic burden have a higher risk of CV events (myocardial infarction and stroke). Given the increased atherosclerotic plaque burden observed in the T2D patients with DR in the present study, we believe that these patients represent a target population in which ultrasound examination of the carotid artery should be performed to evaluate the presence/absence of atherosclerotic lesions in this location. In our opinion, given the association between the atherosclerotic plaque burden and the future risk of CV events shown in general-population studies, a more careful CV assessment and follow-up should be considered in patients with carotid plaques at ultrasound examination.

## Abbreviations

CCA: Common carotid artery
cIMT: carotid intima-media thickness
CKD: Chronic kidney disease
CV: Cardiovascular
CVD: Cardiovascular disease
DR: Diabetic retinopathy
eGFR: Estimated glomerular filtration rate
ICA: Internal carotid artery
VV: Vasa vasorum
