# Correction to: Type 2 diabetes-associated carotid plaque burden is increased in patients with retinopathy compared to those without retinopathy

**DOI:** 10.1186/s12933-018-0696-x

**Published:** 2018-04-05

**Authors:** Núria Alonso, Alicia Traveset, Esther Rubinat, Emilio Ortega, Nuria Alcubierre, Jordi Sanahuja, Marta Hernández, Angels Betriu, Carmen Jurjo, Elvira Fernández, Didac Mauricio

**Affiliations:** 1 Department of Endocrinology and Nutrition, Health Sciences Research Institute and University Hospital Germans Trias i Pujol, Badalona, Spain; 2 Department of Ophthalmology, Hospital Universitari Arnau de Vilanova, Lleida, Spain; 3 Department of Endocrinology and Nutrition, Hospital Universitari Arnau de Vilanova, Lleida, Spain; 4 Institut de Recerca Biomedica de Lleida, University of Lleida, Lleida, Spain; 5 Department of Endocrinology and Nutrition, Institut d’Investigacions Biomediques August Pi Suñer, CIBER de Diabetes y Enfermedades Metabólicas asociadas, Hospital Clinic, 08036 Barcelona, Spain; 6 Unitat de Detecció i Tractament de Malalties Aterotrombòtiques, Hospital Universitari Arnau de Vilanova, Lleida, Spain; 7 Department of Neurology, Hospital Universitari Arnau de Vilanova, Lleida, Spain; 8 Department of Nephrology, Hospital Universitari Arnau de Vilanova, Lleida, Spain

## Correction to: Cardiovasc Diabetol (2015) 14:33 10.1186/s12933-015-0196-1

The author found errors in Table 1 after publication of the original article [1].

The corrected Table 1 is presented in this erratum.

**Table 1:** Characteristics of the study groups

	Diabetes without retinopathy (n = 159)	Diabetic with retinopathy (n = 153)	*p*
Sex—men, n (%)	82 (51.6%)	76 (49.7%)	0.824
Non caucasian, n (%)	7 (4.40%)	7 (4.58%)	1.000
Age (years)	59.0 [48.5;66.0]	61.0 [54.0;68.0]	0.012
Disease duration (years)	6.00 [2.50;10.0]	11.0 [6.00;20.0]	< 0.001
Insulin treatment, n (%)	20 (12.6%)	84 (54.9%)	< 0.001
Smoking (current/past/never) (n)	32/55/71	30/44/79	0.442
Antiplatelet treatment, n (%)	47 (29.6%)	71 (46.4%)	0.003
Dyslipidaemia, n (%)	67 (42.1%)	78 (51.0%)	0.147
Hypertension, n (%)	80 (50.3%)	103 (67.3%)	0.003
Systolic BP (mmHg)	134 [123;145]	143 [133;159]	< 0.001
Diastolic BP (mmHg)	76.0 [70.5;83.0]	77.5 [68.8;85.2]	0.752
Heart rate (b/min)	75.0 [68.0;82.0]	77.0 [70.0;86.0]	0.076
Body mass index (kg/m^2^)	30.3 [27.4;34.0]	31.1 [28.3;35.0]	0.247
Waist (cm)	103 [96.8;111]	107 [101;114]	0.007
Haemoglobin A1c (%)	7.00 [6.50;7.90]	8.10 [7.20;9.10]	< 0.001
Total-cholesterol (mg/dL)	184 [163;206]	181 [163;204]	0.988
HDL-cholesterol (mg/dL)	47.0 [40.0;57.0]	50.5 [42.0;60.2]	0.03
LDL-cholesterol (mg/dL)	109 [90.4;130]	105 [86.5;128]	0.268
Triglycerides (mg/dL)	118 [89.5;169]	116 [83.0;168]	0.617
Creatinine (mg/dL)	0.79 [0.69;0.92]	0.79 [0.68;0.93]	0.797
Urinary albumin/creatinine ratio (mg/g)	5.80 [3.19;10.9]	12.4 [6.00;32.7]	< 0.001

Data are presented as the median values and interquartile ranges

*BP* blood pressure
